# Mental Health of Refugees and Non-refugees from War-Conflict Countries: Data from Primary Healthcare Services and the Norwegian Prescription Database

**DOI:** 10.1007/s10903-016-0450-y

**Published:** 2016-06-21

**Authors:** Melanie L. Straiton, Anne Reneflot, Esperanza Diaz

**Affiliations:** 1 Division of Mental Health, Norwegian Institute of Public Health, PO Box 4404, 0403 Nydalen, Oslo, Norway; 2 Department of Global Public Health and Primary Health Care, University of Bergen, Bergen, Norway

**Keywords:** Immigrant health, Mental health, Refugees, Health care services, Primary health care, Psychotropic medicine

## Abstract

High rates of mental health problems are consistently found among immigrants from refugee generating countries. While refugees and their family members may have experienced similar traumas, refugees are more likely to have undergone a stressful asylum period. This study aims to determine whether their mental health differs. Using national registry data, refugees and non-refugees from the same countries were compared on primary healthcare service use for mental health problems and purchase of psychotropic medicine. Refugees had higher odds of using primary health care services than non-refugees. Refugee women were more likely to purchase psychotropic medicine than non-refugee women. Refugee men were more likely to purchase anti-depressants. The findings suggest that refugees have poorer mental health than non-refugees. This may be due to a combination of greater pre-migration trauma and post-migration stressors such as enduring a difficult asylum period.

## Introduction

Recent years have seen a growth in the number of people seeking asylum around the world. In 2014, there were 714,300 asylum applications in Europe, almost 50 % more than in 2013 [1]. Around 45 % of applicants are granted asylum in the first instance [2]. In 2015, Norway received over 31,000 applications for asylum [3]. By the end of 2014, there were 137,950 refugees living in Norway, and a further 50,180 spouses, children or parents reunited with their refugee counterparts [4]. These groups make up 28.1 % of the immigrant population.

When permission to stay has been granted, all immigrants are covered by the national health insurance scheme and entitled to the same health care as Norwegians. Asylum seekers are now also entitled to this upon arrival [5]. High rates of mental health problems, such as post-traumatic stress, depression and anxiety have been consistently found among those with a refugee background [6, 7], presenting a challenge to the health service as the number of refugees grow. Yet, research on rates of health service use for mental health problems is limited. Small scale studies, often focusing on one specific ethnic group, suggest a low rate of service use compared with need [8–10]. Rates vary considerably by country of origin [11]. In larger scale studies, service use among immigrants from refugee-generating countries (RGCs) is compared to the general population. For instance, in Australia, immigrants from RGCs had lower rates of specialist mental health service use compared with the general population [12], while in Denmark, rates were higher [13]. We lack large scale studies at the primary care level, where majority of mental health problems are treated [14].

Immigrants from RGCs may have experienced traumas such as war, violence, famine, torture, repression and loss of family. These factors are related to increased risk of mental health problems [15]. Those who come as refugees however, are more likely to have endured a difficult and life-threatening migration, in addition to a long, stressful and uncertain period in an asylum centre [16]. This, together with worries about the family left behind may mean that refugees have greater mental health care needs than their later arriving family members. Yet, few studies compare health service use for refugees and non-refugees from the same countries.

A Swedish study compared refugees and non-refugees from RGCs, on the purchase of psychotropic medicine [17]. Refugee women, but not men, were more likely than non-refugees to purchase psychotropic medicine. The authors argued that refugee women were at higher risk of mental health problems than non-refugee women. However, to purchase psychotropic medicine implies not only that one has a mental health problem, but also that one has sought help and thus, differences may also reflect willingness to seek help and/or access to health services.

The current study considers differences in use of primary health care services and psychotropic medicine for mental health problems between refugees and non-refugees from the same RGCs, living in Norway. We have two main hypotheses:

1. Refugees are more likely to use primary health services for mental health problems than non-refugees, even after controlling for socio-demographic factors and general health service use.
2. Focusing only on those who have used primary health care services for mental health problems (and thereby eliminating variation in help-seeking), refugees will be more likely to use psychotropic medicine than non-refugees, even after accounting for socio-demographic factors.

## Method

The National Population Register (NPR), the Norwegian Health Economics Administration database (HELFO) and the Norwegian Prescription Database (NorPD) were linked together for 2008. This was done at an individual level using the personal identification number which all Norwegians at birth and all immigrants living in Norway for more than 6 months are assigned.

NPR provides demographic information. We selected out immigrants from Iraq (N = 13,607), Somalia (N = 11,289), Bosnia and Herzegovina (N = 10,410), Iran (N = 7203), Kosovo (N = 6302) and Afghanistan (N = 4873) aged 20–67 years who had moved to Norway from 1990 onwards. These were the six largest groups from RGCs in 2008. Immigrants were defined as individuals born outside of Norway with two foreign-born parents. Asylum is granted in Norway to those who with good reason to fear persecution in their native country according the Geneva Convention. Although many immigrants from RGCs could apply for residence on these grounds, those who have a spouse, child or parent refugee already living in Norway may alternatively seek residence based on family reunification grounds.

GPs’ compensation claims for all patient contacts within primary health care services (both for GP and emergency primary care consultations) in Norway are made through HELFO. There are different codes for different types of diagnoses and procedures. Diagnoses are recorded based on the International Classification of Primary Care (ICPC-2). We used ICPC-2 codes to determine whether immigrants had ever had a psychological diagnosis during a consultation (P-consultation) in 2008, the number of other, non-P-consultations.

NorPD contains a complete record of all prescribed medicine (from both primary and secondary care) issued from pharmacies. The Anatomical Therapeutic Chemical Classification System is used to categorise drugs based on their therapeutic or chemical characteristics. Anti-psychotics (N05A), anxiolytics (N05B), hypnotics and sedatives (N05C) and anti-depressives (N06A) were extracted from this register.

Ethical approval for the main study, “Immigrants’ health in Norway”, was granted by the Norwegian Data Inspectorate and the Regional committee for Medical and Health Research Ethics, as well as the Norwegian Labour Welfare Service and the Norwegian Directorate of Health. The Norwegian Social Science Data Service prepared the anonymous dataset.

Table 1 gives an overview of the variables used in the study, how they were categorised and the registry they were derived from.

**Table 1:** Overview of variables use and registry source

Variable type	Variable name	Registry	Details	Values
Dependent	P-consultation	HELFO	At least one consultation with psychological diagnosis in 2008 (based on ICPC-2 codes)	Yes/No
	Any psychotropic medicine	NorPD	Purchased any psychotropic medicine, regardless of amount/number of times	Yes/No
	Anti-psychotics drugs	NorPD	Purchased anti-psychotics drugs, regardless of amount/number of times	Yes/No
	Anxiolytics	NorPD	Purchased anxiolytics, regardless of amount/number of times	Yes/No
	Hypnotics & sedatives	NorPD	Purchased hypnotics or sedatives, regardless of amount/number of times	Yes/No
	Anti-depressives	NorPD	Purchased anti-depressives, regardless of amount/number of times	Yes/No
Independent	Refugee status	NPR	Those granted asylum categorised as refugees, all others as non-refugees. Majority of non-refugees granted residence based on family reunification (89 %).	Refugee/Non-refugee
Control variables				
Model 1	Age group	NPR	In years	20−29, 30−39, 40−49, 50+
Model 2	Marital status	NPR	Previously married includes those who are divorced, separated or widowed	married, never married, previously married
	Residence	NPR	Based on proximity to a major city	Urban/non-urban
	Income level	NPR	Work-related income: Low: 1–2,000,000 Norwegian kroner (NOK), medium (200,000–350,000 NOK), high >350000NOK	No, Low, medium, high
	Length of stay	NPR		<5 years, 5–9 years, 10+ years
	Country of origin	NPR		Iraq, Somalia, Bosnia and Herzegovina, Iran, Kosovo, Afghanistan
Model 3	General service use	HELFO	Number of non-P-consultations	1, 2–3, 4+

### Statistical Analyses

To address the first hypothesis, we conducted Chi square analyses and then logistic regression analyses while controlling for age group (model 1) and then other socio-demographics (marital status, residence, income level, country of origin and length of stay) (model 2). In model 3 we added in number of non-P-consultations as a measure of general service use). For the second hypothesis, we selected out all immigrants who had had at least one P-consultation and conducted Chi square analyses. Logistic regression analyses for each type of psychotropic medicine were also carried out, adjusting for age-group in model 1, and marital status, income level, country of origin and length of stay in model 2.

Analyses were conducted in SPSS version 20, separately for men and women.

## Results

The total sample included 53,747 immigrants; 54.5 % were men. Men were more often refugees than women (82.0 % vs 59.0 % (*X* ^2^ = 3449.8, df = 1, *p* < 0.05)), though percentages varied by country of origin. This is shown in Table 2, together with an overview of demographics. Refugee men were older than non-refugee men and were more often married. They were less likely to have urban residence, no or a low income and a short length of stay (<5 years). Refugee women were also older than non-refugee women and less likely to have urban residence, no or a low income and a short length of stay (<5 years). Refugee women were less likely to be married than non-refugee women.

**Table 2:** Overview of demographic variables and psychological (P-) consultations for refugees and non-refugees

	Men		Women	
	Non-refugees	Refugees	Non-refugees	Refugees
	(N = 5285) %	(N = 24,022) %	(N = 10,019) %	(N = 14,421) %
Average age years (SD)	32.4 (10.5)*	37.2 (10.7)	33.2 (9.1)*	37.5 (11.5)
Married	47.3*	58.4	73.1*	55.0
Urban residence	83.7*	80.4	84.8*	82.0
No/Low income	53.7*	49.5	78.5*	66.1
Length of stay < 5 years	39.4*	15.8	38.3*	14.6
% refugees from each country^+^				
Iraq		84.8		35.9
Somalia		76.5		61.1
Bosnia-Herzegovina		88.6		85.8
Iran		73.5		51.2
Kosovo		79.4		67.9
Afghanistan		87.2		48.4
Had P-consultation	10.2*	15.7	12.3*	19.0

* Significant difference between refugees and non-refugees (*p* < 0.05)

^+^Significant overall differences in percentage of refugees by country of origin (*p* < 0.05)

### P-consultations

Crude rates of P-consultations are also shown in Table 2. For both men and women, a higher percentage of refugees had had at least one P-consultation compared with non-refugees. Table 3 shows refugees’ odds of having had a P-consultation compared with non-refugees after accounting for age (model 1), other socio-demographics (model 2) and general service use (model 3). Refugees had significantly higher odds of having a P-consultation than non-refugees for both men and women in all models.

**Table 3:** Adjusted odds of P-consultation for men and women

	Men			Women		
	Model 1^a^	Model 2^b^	Model 3^c^	Model 1^a^	Model 2^b^	Model 3^c^
	OR (95 % CI)	OR (95 % CI)	OR (95 % CI)	OR (95 % CI)	OR (95 % CI)	OR (95 % CI)
*Refugee status*						
Non-refugees	1.00	1.00	1.00	1.00	1.00	1.00
Refugees	1.53 (1.39–1.68)*	1.35 (1.22–1.49)*	1.29 (1.17–1.43)*	1.52 (1.41–1.63)*	1.48 (1.36–1.61)*	1.43 (1.32–1.56)*
*Age group*						
20–29	1.00	1.00	1.00	1.00	1.00	1.00
30–39	1.35 (1.24–1.47)*	1.27 (1.14–1.40)*	1.27 (1.15–1.41)*	1.44 (1.32–1.58)*	1.35 (1.21–1.49)*	1.35 (1.22–1.50)*
40–49	1.75 (1.61–1.91)*	1.41 (1.26–1.58)*	1.39 (1.24–1.56)*	2.04 (1.86–2.24)*	1.66 (1.49–1.86)*	1.69 (1.51–1.89)*
50+	1.83 (1.65–2.03)*	1.20 (1.05–1.37)*	1.11 (0.97–1.27)	1.91 (1.71–2.13)*	1.41 (1.23–1.61)*	1.39 (1.22–1.60)*
*Marital status*						
Married		1.00	1.00		1.00	1.00
Never married		1.02 (0.93–1.13)	1.13 (1.03–1.24)*		1.13 (1.01–1.27)*	1.27 (1.13–1.42)*
Previously married		1.65 (1.50–1.83)*	1.73 (1.56–1.91)*		1.77 (1.62–1.94)*	1.79 (1.64–1.96)*
*Residence*						
Urban		1.00	1.00		1.00	1.00
Sub-urban/rural		0.89 (0.81–0.97)*	0.90 (0.82–0.99)*		0.96 (0.87–1.06)	0.98 (0.89–1.08)
*Income (nok)*						
No income		2.03 (1.84–2.23)*	2.27 (2.05–2.50)*		1.14 (1.03–1.26)*	1.22 (1.11–1.36)*
Low		1.30 (1.19–1.43)*	1.39 (1.26–1.52)*		1.17 (1.05–1.29)*	1.21 (1.10–1.34)*
Medium		1.00	1.00		1.00	1.00
High		0.70 (0.63–0.78)*	0.73 (0.66–0.81)*		0.92 (0.80–1.05)	0.97 (0.85–1.12)
*Country of origin*						
Iraq		1.00	1.00		1.00	1.00
Somalia		0.47 (0.42–0.52)*	0.48 (0.43–0.54)*		0.33 (0.29–0.38)*	0.33 (0.29–0.38)*
Bosnia-H		0.91 (0.82–1.02)	0.97 (0.87–1.09)		0.90 (0.80–1.02)	0.99 (0.88–1.12)
Iran		1.13 (1.01–1.36)*	1.15 (1.03–1.28)*		1.55 (1.39–1.73)*	1.60 (1.43–1.78)*
Kosovo		0.96 (0.86–1.08)	0.98 (0.87–1.10)		1.16 (1.03–1.31)*	1.21 (1.07–1.37)*
Afghanistan		1.03 (0.91–1.16)	1.07 (0.94–1.21)		1.37 (1.19–1.57)*	1.38 (1.19–1.59)*
*Length of stay*						
<5 years		0.61 (0.54–0.68)*	0.63 (0.56–0.71)*		0.57 (0.51–0.65)*	0.59 (0.52–0.66)*
5–9 years		0.93 (0.86–1.01)	0.90 (0.83–0.98)*		0.93 (0.86–1.02)	0.91 (0.84–1.00)
10+ years		1.00	1.00		1.00	1.00
*General service use (non-P-consultation)*						
0 consultations			1.00			1.00
1–2 consultations			1.71 (1.57–1.88)*			1.63 (1.44–1.84)*
3–5 consultations			2.07 (1.88–2.28)*			2.28 (2.02–2.57)*
>6 consultations			2.33 (2.10–2.58)*			2.45 (2.18–2.76)*

* (*p* < 0.05)

^a^Adjusting for age

^b^Adjusting for age, marital status, urban residence, income level, country of origin and length of stay

^c^Adjusting for age, marital status, urban residence, income level, country of origin, length of stay and general service use

All socio-demographic variables (except residence for women) related to odds of P-consultations too, and in similar ways for men and women. Odds of consultation were higher for those aged 40–49 years, those who had previously been married and those with no or low incomes. For men, high income and suburban/rural residence was associated with lower odds of P consultation. Shorter length of stay was associated with lower odds for both men and women. Compared with immigrants from Iraq, immigrants from Somali had far lower odds of a P-consultation while immigrants from Iran had higher odds. Afghan and Kosovar women also had higher odds than Iraqi women. Higher general service use was associated with higher odds of P-consultation.

### Purchased Psychotropic Medicine

**Fig. 1:**
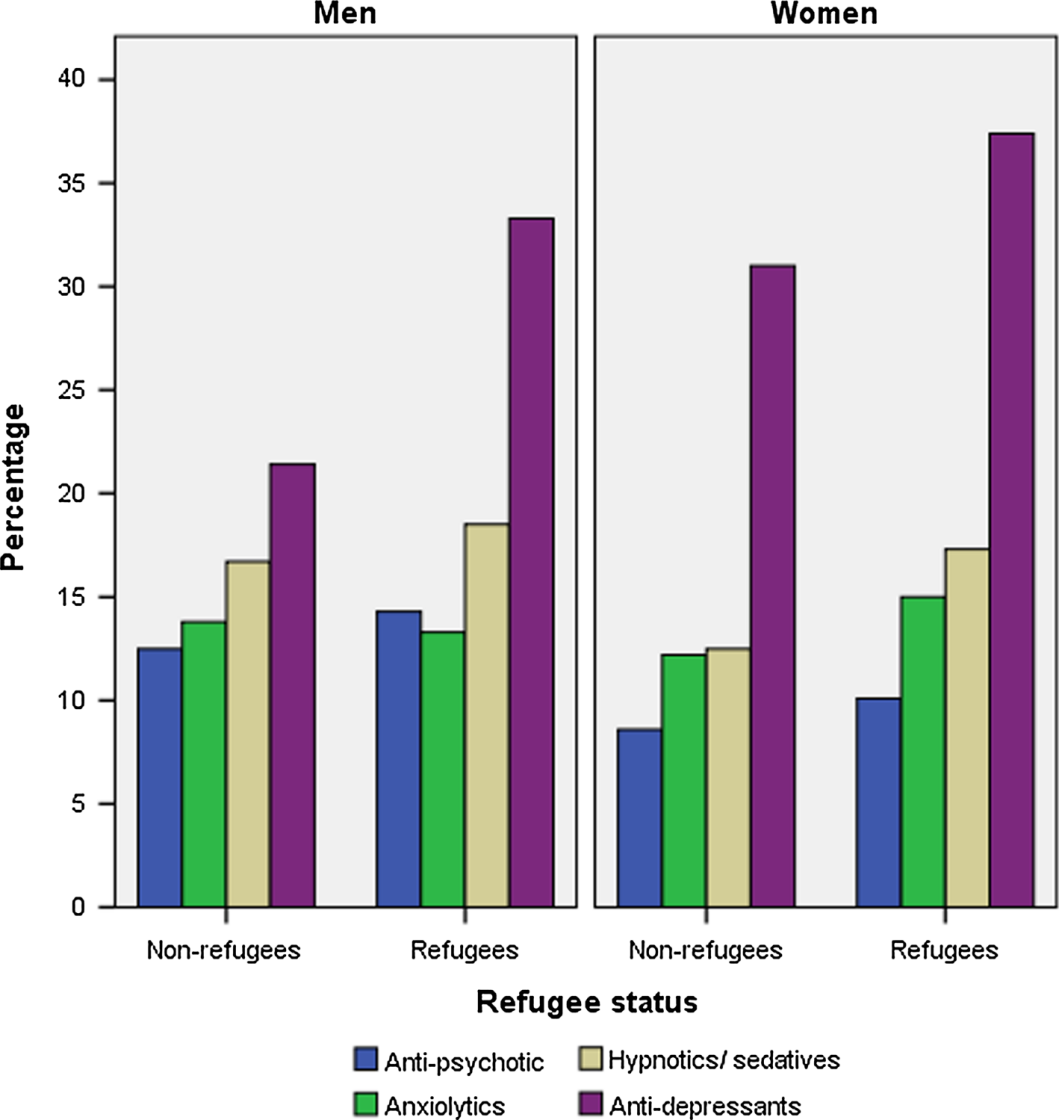
Percentage of refugees and non-refugees who have purchased psychotropic medicine by type

To address the second research question, we selected out all immigrants who had had at least one P-consultation in 2008 (4303 men and 3979 women). Refugee men were more likely to have purchased at least one type of psychotropic medicine than non-refugee men (50.9 vs. 43.1 %, *X* ^2^ = 10,34, df = 1, *p* < 0.05). A similar finding was noted for women (refugees: 54.0 vs. 45.8 % for refugees and non-refugees respectively; *X* ^2^ = 27.91, df = 1, *p* < 0.05). Crude rates of psychotropic medicine by type are shown in Fig. 1. For men, the only significant difference was on anti-depressants, with a higher percentage of refugees purchasing anti-depressants than non-refugees (*X* ^2^ = 30.52, df = 1, *p* < 0.05). For women, refugees were more likely to have purchased anxiolytics (*X* ^2^ = 5,25, df = 1, *p* < 0.05), hypnotics/sedatives (*X* ^2^ = 14,68, df = 1, *p* < 0.05) and anti-depressants (*X* ^2^ = 15,52, df = 1, *p* < 0.05) than non-refugees.

Table 4 shows the findings for the multivariate analyses. Refugee and non-refugee men no longer differed on purchase of psychotropic drugs and refugee and non-refugee women no longer differed on purchase of anxiolytic drugs after adjusting for age in model 1. The addition of other demographic variables in model 2 did not fully explain the remaining differences.

**Table 4:** Odds ratio (OR) and 95 % confidence intervals (CI) for refugees’ purchase of psychotropic medicine compared with non-refugees

	Model 1^a^	Model 2^b^
	OR (95 % CI)	OR (95 % CI)
*Men*		
Psychotropic medicine (any)	1.14 (0.94–1.38)	1.03 (0.85–1.26)
Anti–psychotics	1.03 (0.78–1.36)	0.90 (0.67–1.20)
Anxiolytics	0.87 (0.66–1.13)	0.75 (0.57–1.00)
Hypnotics	0.95 (0.74–1.22)	0.95 (0.74–1.23)
Anti-depressants	1.52 (1.22**–**1.90)*	1.36 (1.08**–**1.71)*
*Women*		
Psychotropic medicine (any)	1.26 (1.10**–**1.45)*	1.19 (1.02**–**1.39)*
Anti**-**psychotics	1.14 (0.90**–**1.44)	1.08 (0.83**–**1.40)
Anxiolytics	1.17 (0.96**–**1.44)	1.03 (0.82**–**1.28)
Hypnotics	1.38 (1.13**–**1.68)*	1.29 (1.04**–**1.60)*
Anti**-**depressants	1.22 (1.05**–**1.41)*	1.21 (1.03**–**1.43)*

* *p* < 0.05

^a^Adjusting for age

^b^Adjusting for age, marital status, income level, country of origin and length of stay

## Discussion

The study shows that refugees are more likely than non-refugees to have used primary health care services for mental health problems. The reason for this is not clear. Refugees may have experienced more pre- and post-migration trauma and have a greater need for mental health care [17]. Alternatively, refugees could be more likely to seek health care than their non-refugee counterparts. As part of the refugee resettlement programme, refugees will gain insight into Norwegian society and learn about the health system [18]. This may facilitate help-seeking. Immigrants who come through family reunification do not necessarily receive the same support. It may be that this somewhat forgotten, at risk group, requires more support and information about available services upon being issued a residence permit. Efforts should be made to increase the flow of health care information, especially for non-refugees who may experience greater barriers. The government needs to ensure that information about health care services is available in multiple languages, accessible and adapted for all new immigrants. Additionally, programmes increasing mental health literacy, which also addressing stigma, a barrier to help-seeking [19], may improve help-seeking among immigrants.

In line with other studies showing that Somalis use health care services less than other immigrant groups [11], we found that Somali men and women were the group least likely to have had a P-consultation. Although Somalis may report better mental health than other refugee groups [20], studies also suggest that they do not access care according to need [9]. Iranian men and women were the group most likely to have had a P-consultation. Iranian immigrants are highly educated compared with some other refugee populations [21]. Although higher education is considered protective for mental health problems [22], education level has also been found to be positively related to help-seeking among those with a mental health problem [23]. Higher education may facilitate help-seeking through greater health literacy and earlier recognition of a problem. However, Iranians were still more likely to have had a P-consultation than other groups after adjustment for socio-demographic variables. It is perhaps more likely then, that Iranian immigrants are at greater risk of mental health problems than other refugee groups, reflecting findings from research in the Netherlands [11, 20].

Immigrants who had been in Norway less than 5 years were less likely to have had a P-consultation than immigrants staying more than 10 years. Health care use may increase through time as an immigrant learns to navigate the health system, or it may be that mental health problems emerge or worsen over time. Indeed, post-migration stress including discrimination, economic difficulties and language difficulties can contribute to mental health problems [15, 24]. It also possible that refugees in particular, receive care through other services such as social work during the resettlement process and thus, do not seek help from their GP in earlier years.

Our second research question addressed the purchase of psychotropic drugs. Selecting out only those who had had a P-consultation, we eliminated differences in help-seeking between refugees and non-refugees. We found that refugee women, but not men, were more likely to purchase any type of psychotropic medicine than their non-refugee counterparts. This supports the suggestion from Hollander et al. [17] who argued that refugee women had poorer mental health than non-refugee women. More specifically, refugee women, in the current study, were more likely to purchase anti-depressants and hypnotics/sedatives than non-refugee women, suggesting that depressive and anxiety disorders/sleep disturbances are more prevalent among refugee than non-refugee women. When considering drug type, we also found that refugee men had higher odds of purchasing anti-depressants than non-refugee men, suggesting that depressive disorders may be more prevalent in this group.

Refugees’ greater risk of depression compared with their non-refugee counterparts may partly be related to the stress associated with the asylum-seeking process. Uncertainty of the future, deprivation and feelings of powerlessness are commonly reported by asylum-seekers [25, 26]. Longer periods in reception centres are associated with greater severity of mental health problems [27]. While recent efforts to increase empowerment among asylum seekers in Norwegian reception centres have been made [26], the impact on mental health is not yet known. An alternative explanation for refugees’ apparent poorer mental health relates to pre-migration factors. While it is likely that immigrants coming from war-conflict countries will have some similar experiences, it may be that those who flee first are in greater danger and have experienced more direct trauma than a family member who joins later. Cumulative exposure to trauma and the experience of torture are strong predictors of subsequent depression among refugees [7] but we were unable to account for pre-migration factors.

This study is one of few comparing refugees with non-refugees from the same RGCs. It benefits from the use of nationwide register data, which does not rely on self-report or recall biases. It uses a reliable and precise way of identifying refugees [28] and the relatively large population allows us to account for differences in country background. Another strength is the elimination of differences in help-seeking for mental health problems when considering psychotropic medicine.

Register data is not without its limitations. Use of HELFO data relies on accurate completion by GPs. Diagnoses are made based on the ICPC-2 for administrative claims and are reliable at the chapter level for investigation purposes [29] but cannot give an indication of the prevalence of particular disorders [30]. It should be noted that data from the prescription register are based only on prescriptions that have been exchanged at a pharmacy and thus do not reflect all psychotropic medicine prescribed. However, there is no reason to believe that non-refugees would exchange prescriptions less often than refugees. In the analysis, we did not account for those who had purchased psychotropic medicine but had not had a P-consultation in 2008. There are likely to be others who have had P-consultations in 2007 who were issued repeat prescriptions.

## Conclusion

The findings of this study might imply that (a) non-refugees may access health services to a lesser extent than refugees, (b) refugees may have greater mental health problems than non-refugees from the same countries due to greater pre- and post-migration stress or (c) a combination of both. Further research is required to determine the reasons. Assuming a combination of both, more resources should be invested in informing non-refugees about available health services to encourage help-seeking and ways of reducing the stress of the asylum period following arrival in Norway should be sought. Regardless, both refugees and non-refugees from RGCs are at risk of mental health problems and both groups are likely to benefit from an increased focus on mental health with early offers of help.
